# Multicenter Phase II Study of Lurbinectedin in *BRCA*-Mutated and Unselected Metastatic Advanced Breast Cancer and Biomarker Assessment Substudy

**DOI:** 10.1200/JCO.2018.78.6558

**Published:** 2018-09-21

**Authors:** Cristina Cruz, Alba Llop-Guevara, Judy E. Garber, Banu K. Arun, José A. Pérez Fidalgo, Ana Lluch, Melinda L. Telli, Cristian Fernández, Carmen Kahatt, Carlos M. Galmarini, Arturo Soto-Matos, Vicente Alfaro, Aitor Pérez de la Haza, Susan M. Domchek, Silvia Antolin, Linda Vahdat, Nadine M. Tung, Rafael Lopez, Joaquín Arribas, Ana Vivancos, José Baselga, Violeta Serra, Judith Balmaña, Steven J. Isakoff

**Affiliations:** Cristina Cruz and Judith Balmaña, Vall d’Hebron Hospital; Cristina Cruz, Alba Llop-Guevara, Joaquín Arribas, Ana Vivancos, Violeta Serra, and Judith Balmaña, Vall d’Hebron Institute of Oncology; José A. Pérez Fidalgo, Ana Lluch, Joaquín Arribas, and Violeta Serra, Centro de Investigación Biomédica en Red; Joaquín Arribas, Institució Catalana de Recerca i Estudis Avançats, Barcelona; José A. Pérez Fidalgo and Ana Lluch, Hospital Clínico de Valencia, Valencia; Cristian Fernández, Carmen Kahatt, Carlos M. Galmarini, Arturo Soto-Matos, Vicente Alfaro, and Aitor Pérez de la Haza, PharmaMar, Madrid; Silvia Antolin, Complejo Universitario Hospitalario La Coruña, La Coruña; Rafael Lopez, Complejo Hospitalario Universitario Santiago de Compostela, Santiago de Compostela, Spain; Judy E. Garber, Dana Farber Cancer Institute; Nadine M. Tung, Beth Israel Deaconess Medical Center; José Baselga and Steven J. Isakoff, Massachusetts General Hospital Cancer Center, Boston, MA; Banu K. Arun, MD Anderson Cancer Center, Houston, TX; Melinda L. Telli, Stanford University School of Medicine, Stanford, CA; Susan M. Domchek, University of Pennsylvania, Philadelphia, PA; and Linda Vahdat, Weill Cornell Medicine, New York, NY.

## Abstract

**Purpose:**

This multicenter phase II trial evaluated lurbinectedin (PM01183), a selective inhibitor of active transcription of protein-coding genes, in patients with metastatic breast cancer. A unicenter translational substudy assessed potential mechanisms of lurbinectedin resistance.

**Patients and Methods:**

Two arms were evaluated according to germline *BRCA1/2* status: *BRCA1/2* mutated (arm A; n = 54) and unselected (*BRCA1/2* wild-type or unknown status; arm B; n = 35). Lurbinectedin starting dose was a 7-mg flat dose and later, 3.5 mg/m^2^ in arm A. The primary end point was objective response rate (ORR) per Response Evaluation Criteria in Solid Tumors (RECIST). The translational substudy of resistance mechanisms included exome sequencing (n = 13) and in vivo experiments with patient-derived xenografts (n = 11) from *BRCA1/2-*mutated tumors.

**Results:**

ORR was 41% (95% CI, 28% to 55%) in arm A and 9% (95% CI, 2% to 24%) in arm B. In arm A, median progression-free survival was 4.6 months (95% CI, 3.0 to 6.0 months), and median overall survival was 20.0 months (95% CI, 11.8 to 26.6 months). Patients with *BRCA2* mutations showed an ORR of 61%, median progression-free survival of 5.9 months, and median overall survival of 26.6 months. The safety profile improved with lurbinectedin dose adjustment to body surface area. The most common nonhematologic adverse events seen at 3.5 mg/m^2^ were nausea (74%; grade 3, 5%) and fatigue (74%; grade 3, 21%). Neutropenia was the most common severe hematologic adverse event (grade 3, 47%; grade 4, 10%). Exome sequencing showed mutations in genes related to the nucleotide excision repair pathway in four of seven tumors at primary or acquired resistance and in one patient with short-term stable disease. In vivo, sensitivity to cisplatin and lurbinectedin was evidenced in lurbinectedin-resistant (one of two) and cisplatin-resistant (two of three) patient-derived xenografts.

**Conclusion:**

Lurbinectedin showed noteworthy activity in patients with *BRCA1/2* mutations. Response and survival was notable in those with *BRCA*2 mutations. Additional clinical development in this subset of patients with metastatic breast cancer is warranted.

## INTRODUCTION

Metastatic breast cancer (MBC) is a heterogeneous disease. Some new therapeutic approaches offer a tailored therapy on the basis of tumor characteristics. Beyond hormone receptor (HR) and human epidermal growth factor receptor 2 (HER2) status, a better knowledge of the DNA damage response pathway has allowed the identification of actionable targets for MBC.

Approximately 3% to 5% of unselected patients with MBC carry a germline mutation in *BRCA1* or *BRCA2* genes. These genes encode for two tumor suppressor proteins essential in homologous recombination repair (HRR), a vital DNA repair process that uses the undamaged sister chromatid to carry out high-fidelity repair of DNA double-strand breaks.^1^ Trabectedin, an antitumor agent that relies on an efficient nucleotide excision repair (NER) and a deficient HRR pathway, has shown remarkable activity in heavily pretreated patients with MBC with a germline *BRCA1/2* mutation.^2-4^

Lurbinectedin, a trabectedin analog, is a selective inhibitor of the active transcription of protein-coding genes. The mechanism involves the irreversible stalling of elongating RNA polymerase II on the DNA template and its specific degradation by the ubiquitin/proteasome machinery. Subsequently, recruitment of DNA repair factors, including XPF nuclease, induces the accumulation of double-strand breaks and apoptosis as downstream events.^5^ These effects are increased in HRR-deficient cells. Indeed, in *BRCA2*-mutated cells, this could be related to the concurrence of deficient DNA repair and formation of R-loops that occurs during the elongation step of transcription by RNA polymerase II.^6,7^

Both antitumor activity observed with lurbinectedin against HRR-deficient cell lines^8,9^ and clinical activity observed with trabectedin prompted the conduct of this phase II trial to evaluate the efficacy and safety of lurbinectedin in patients with MBC with deleterious germline *BRCA1/2* mutations or unselected disease. Two arms were evaluated according to germline *BRCA1/2* status: *BRCA1/2* mutated (arm A) and unselected (*BRCA1/2* wild-type or unknown status; arm B). In parallel, a correlative translational research study was undertaken to identify predictive biomarkers of response and resistance through exome sequencing of patients’ biopsy samples and in vivo efficacy analyses in preclinical models.

## PATIENTS AND METHODS

Patients were recruited from 11 investigational sites in the United States and Spain. The study protocol was approved by the independent local ethics committee at each participating center and was conducted in accordance with the Declaration of Helsinki, Good Clinical Practice guidelines, and local regulations for clinical trials. Signed informed consent was obtained from all patients before any study-specific procedure.

### Eligibility Criteria

Patients eligible for this study were 18 to 75 years old with a histologically proven diagnosis of MBC, no more than three prior chemotherapy-containing regimens in the advanced setting (including at least one prior trastuzumab-containing regimen in those with known HER2-overexpressing tumors), measurable disease per Response Criteria in Solid Tumors (RECIST) version 1.1,^10^ Eastern Cooperative Oncology Group performance status ≤ 1, and adequate major organ function. Patients were excluded if they had previously received lurbinectedin, trabectedin, or radiotherapy to > 35% of bone marrow; prior or concurrent other malignant disease unless in complete remission for > 5 years; symptomatic, corticosteroid-requiring, or progressive CNS involvement; exclusively bone-limited disease; concomitant unstable or serious medical condition, or impending need for radiotherapy.

### Treatment

All patients were treated with lurbinectedin administered as a 1-hour intravenous (IV) infusion every 3 weeks. The starting dose was a 7.0-mg flat dose (FD) on the basis of results from the first-in-human phase I trial.^11^ On the basis of new (unpublished) data from this and other lurbinectedin studies that suggested body surface area (BSA)–related toxicity, the study was amended to introduce BSA-based dosing. After an amendment, patients received lurbinectedin as a 1-hour IV infusion on day 1 every 3 weeks at a starting dose of 3.5 mg/m^2^ (capped at BSA 2.0 m^2^). All patients received antiemetic prophylaxis. Granulocyte colony-stimulating factors were allowed for secondary prevention of neutropenia.

### Efficacy Assessment

The primary efficacy end point was objective response rate (ORR) according to RECIST version 1.1. Secondary efficacy end points were duration of response, clinical benefit (ORR or stable disease > 3 months), progression-free survival (PFS), and overall survival (OS). Patients evaluable for efficacy received at least one complete lurbinectedin infusion and had at least one tumor assessment. Radiologic tumor evaluation was performed every 6 weeks (two cycles) until cycle six, and every 9 weeks (three cycles) thereafter. Objective response was confirmed at least 4 weeks later. Patients in arm B who achieved a confirmed response and whose *BRCA1/2* status was unknown were tested for *BRCA1/2* germline variants.

### Safety Assessment

Safety was evaluated in all patients who received at least one lurbinectedin infusion by assessment of adverse events (AEs), clinical laboratory test results, physical examinations, and vital signs. AEs were recorded and coded with the Medical Dictionary for Regulatory Activities version 12.0. AEs and laboratory values were graded according to the National Cancer Institute Common Toxicity Criteria for Adverse Events (version 4.0). All patients were followed until recovery from any lurbinectedin-related AE.

### Pharmacogenomic Substudy

Single nucleotide polymorphisms and DNA mutations in a panel of 151 cancer-related genes were centrally analyzed (Appendix, online only).

### Fresh Biopsy Cohort, Exome Sequencing, Implantation of Patient-Derived Xenograft Models, and In Vivo Experiments

All patients from one of the investigational sites were offered to participate in a translational substudy (Appendix).

### Statistical Methods

A futility analysis on the basis of the primary end point (ORR) was planned after 20 and 30 evaluable patients had been treated in arms A and B, respectively. If fewer than four of the 20 patients in arm A or fewer than three of the 30 patients in arm B achieved an objective confirmed response, recruitment into that arm was halted. Otherwise, recruitment was planned until at least 53 and 64 evaluable patients were included in each arm, respectively (Appendix).

In the clinical part of the study, binomial exact estimator and 95% CI were calculated for ORR. Kaplan-Meier method was used to analyze PFS and OS (compared in subgroups by unstratified log-rank test). Logistic regression was used in the ORR multivariable analysis.

Statistical analysis for in vitro data consisted of *t* test or one-way analysis of variance (Dunnett’s post hoc test). Two-way analysis of variance (Tukey’s post hoc test) was used for in vivo patient-derived xenograft (PDX) data. Multiplicity-adjusted *P* values are reported, and statistical significance was achieved if *P* < .05.

## RESULTS

### Patient Characteristics

**Fig 1.**
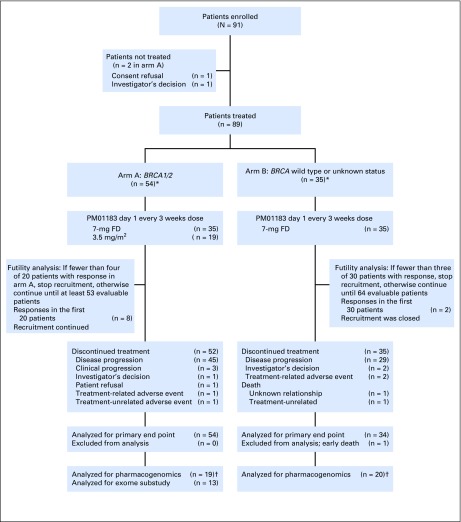
CONSORT diagram for both the phase II clinical trial and the biomarker substudy. (*) Per study design, recruitment had to continue until at least 53 patients in arm A (complete cohort) and 30 patients in arm B (futility analysis) were evaluable for response. First tumor assessment in each patient took place per protocol 6 weeks after first lurbinectedin infusion. At that time, once the required 53 and 30 evaluable patients had been included, one additional patient in arm A and five additional patients in arm B also had been enrolled. (†) The sample size in pharmacogenomic analyses corresponds to samples and not to number of patients. FD, flat dose.

Eighty-nine patients were treated with lurbinectedin between June 2012 and March 2016 (arm A, 54 patients; arm B, 35 patients; Fig 1). In arm A, 57% and 43% of patients carried deleterious *BRCA1* and *BRCA2* mutations, respectively; 56% and 44% had triple-negative and HR+ disease; and 59% had more than two tumor sites (liver metastasis, 52%; CNS metastasis, 6%). Patients received a median of one (range, zero to three) prior advanced chemotherapy lines, including platinum in 37% (19% in the adjuvant/neoadjuvant setting; Table 1).

**Table 1.**
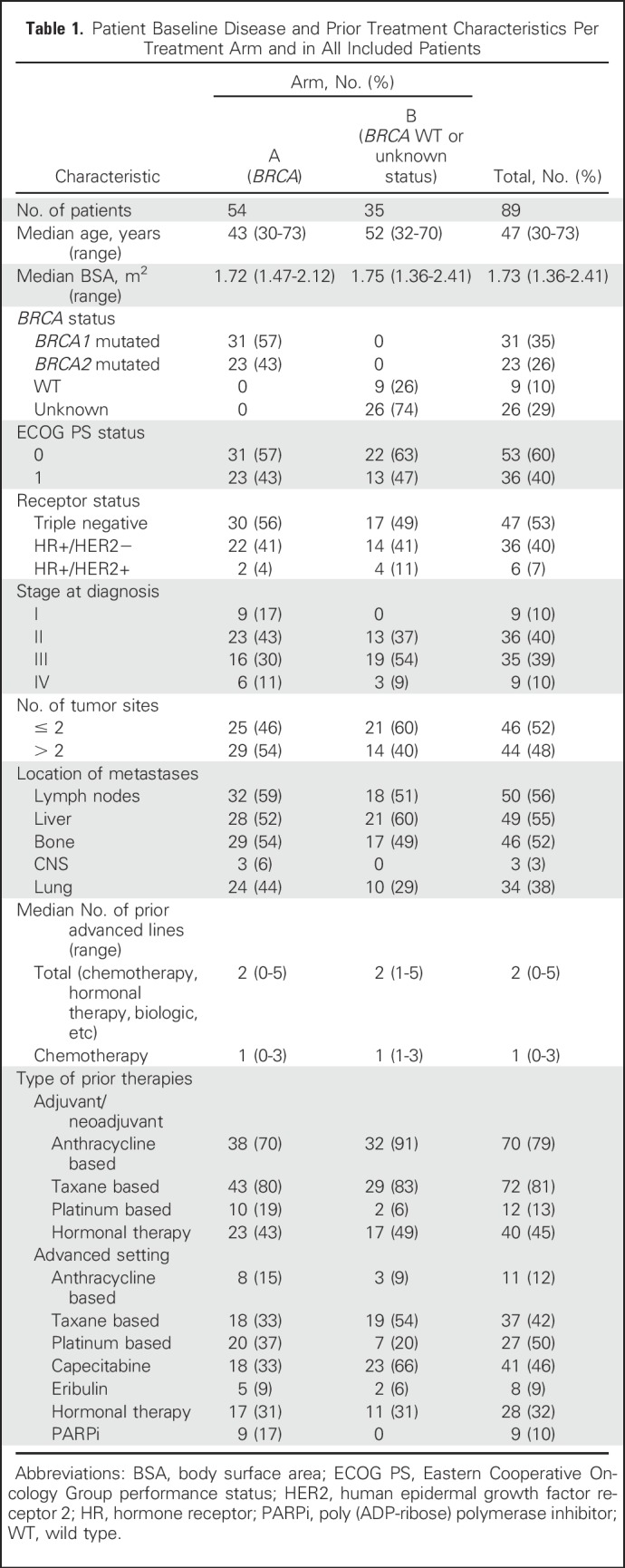
Patient Baseline Disease and Prior Treatment Characteristics Per Treatment Arm and in All Included Patients

In arm B, 74% of patients had unknown *BRCA1/2* status (26% were *BRCA1/2* negative); 49% and 51% had triple-negative and HR+ disease, respectively; and 40% had more than two tumor sites (liver metastasis, 60%). The median number of advanced chemotherapy lines was one (range, one to three), and 6% and 20% of patients received prior platinum-based chemotherapy in the adjuvant/neoadjuvant and advanced setting, respectively (Table 1). No relevant differences between arms were observed (Appendix Table A1, online only).

### Dosing

In arm A, 35 patients received lurbinectedin at a starting dose of 7 mg FD (median of six cycles per patient [range, one to 24 cycles]), and 19 patients received 3.5 mg/m^2^ (median of nine cycles per patient [range, two to 30 cycles]). In arm B, all 35 patients received lurbinectedin at 7 mg FD (median of three cycles [range, one to 27 cycles]; Fig 1).

### Efficacy in Arm A (*BRCA1/2* Mutated)

**Fig 2.**
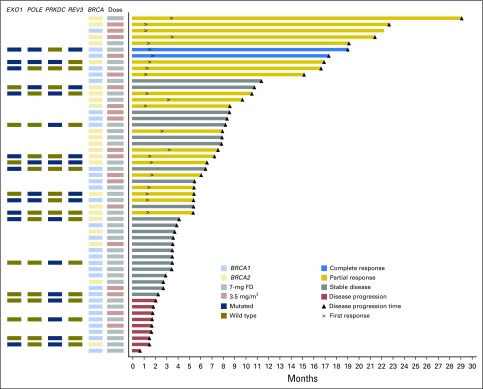
Swimmer plot that shows clinical response, duration of therapy (in months), *BRCA* status, and lurbinectedin dose received in patients from arm A (*BRCA1/2* mutated). Each bar represents one patient in the study (n = 54). Information from pharmacogenomic analyses is shown in the columns located to the left. FD, flat dose.

**Fig A1.**
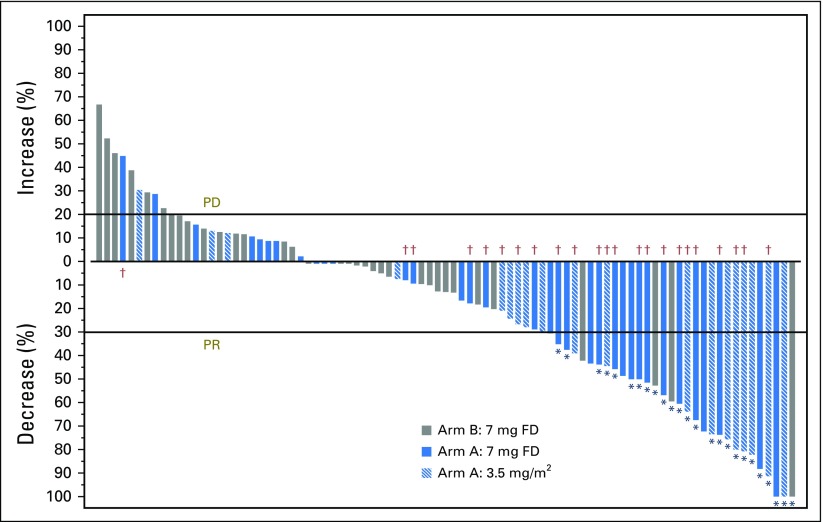
Efficacy of lurbinectedin by dose. A waterfall plot shows the maximum variation of target lesions in patients with at least one radiologic tumor assessment in arm A (*BRCA1/2*) and arm B (*BRCA* wild-type or unknown status). One patient in arm B had a complete response in target lesions but not in nontarget lesions (objective response was then partial). (*) Confirmed response. (†) Patients with *BRCA2* mutation. FD, flat dose; PR, partial response; PD, disease progression.

**Fig A2.**
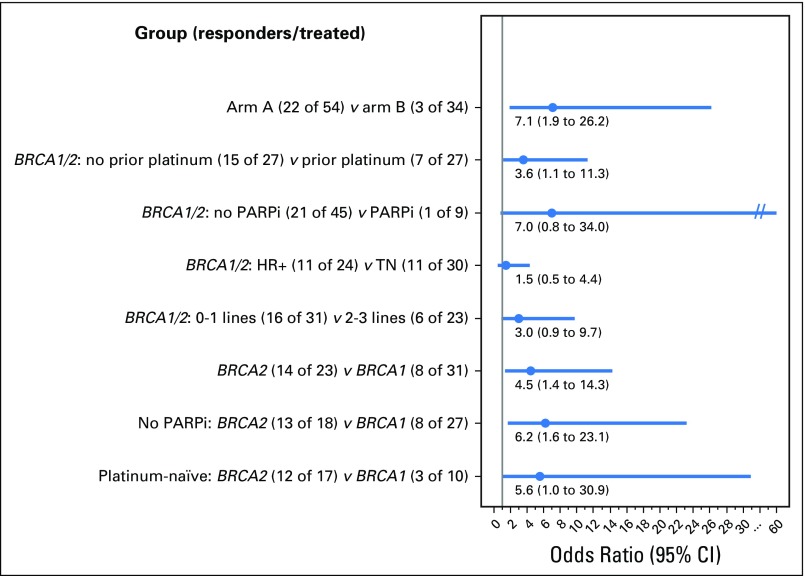
Summary of odds ratios and 95% CIs for objective response rate in the subgroups analyzed. Data shown include responder and treated patients. HR, hormone receptor; PARPi, poly (ADP-ribose) polymerase inhibitor; TN, triple negative.

Futility analysis conducted on the first 20 evaluable patients showed eight objective responses (ORR, 40.0%; 95% CI, 19% to 64%); therefore, recruitment continued to accrue 54 evaluable patients. ORR at final analysis was 41% (95% CI, 28% to 55%), including two complete responses (CRs) and 20 partial responses (PRs; Table 2; Appendix Fig A1, online only). Efficacy was not affected by lurbinectedin dose modification from 7 mg FD to 3.5 mg/m^2^ (Fig 2). Median duration of response was 6.1 months (95% CI, 3.4 to 11.3 months). Disease control rate and clinical benefit rate were 83% and 61%, respectively. Responding patients had received a median of one prior advanced chemotherapy line (range, zero to two lines). Prespecified subgroup analysis showed an ORR of 61% (95% CI, 38.5% to 80.3%) in patients with the *BRCA*2 mutation and 26% (95% CI, 11.9% to 44.6%) in those with the *BRCA1* mutation. ORR increased in patients without prior poly (ADP-ribose) polymerase inhibitor (PARPi) therapy, especially in *BRCA2* (ORR, 72% *v* 30% in *BRCA1*). ORR in patients with HR+ versus triple-negative disease was 46% (95% CI, 25.6% to 67.2%) and 37% (95% CI, 19.9% to 56.1%), respectively. With regard to prior platinum, ORR was 26% (95% CI, 11.1% to 46.3%) and 56% (95% CI, 35.3% to 74.5%) for patients with or without prior platinum therapy, respectively. Among platinum-naïve patients (n = 27), ORR in *BRCA1* and *BRCA2* was 30% and 71%, respectively. ORR odds ratio per subgroup is shown in Appendix Fig A2 (online only). Multivariable analysis including *BRCA* status, HR, and prior platinum and advanced chemotherapy lines showed *BRCA2* as the only significant variable for predicting response.

**Table 2.**
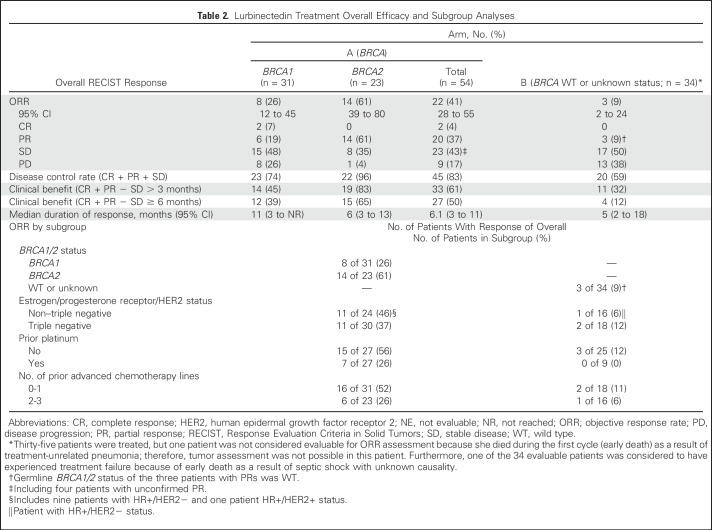
Lurbinectedin Treatment Overall Efficacy and Subgroup Analyses

**Fig 3.**
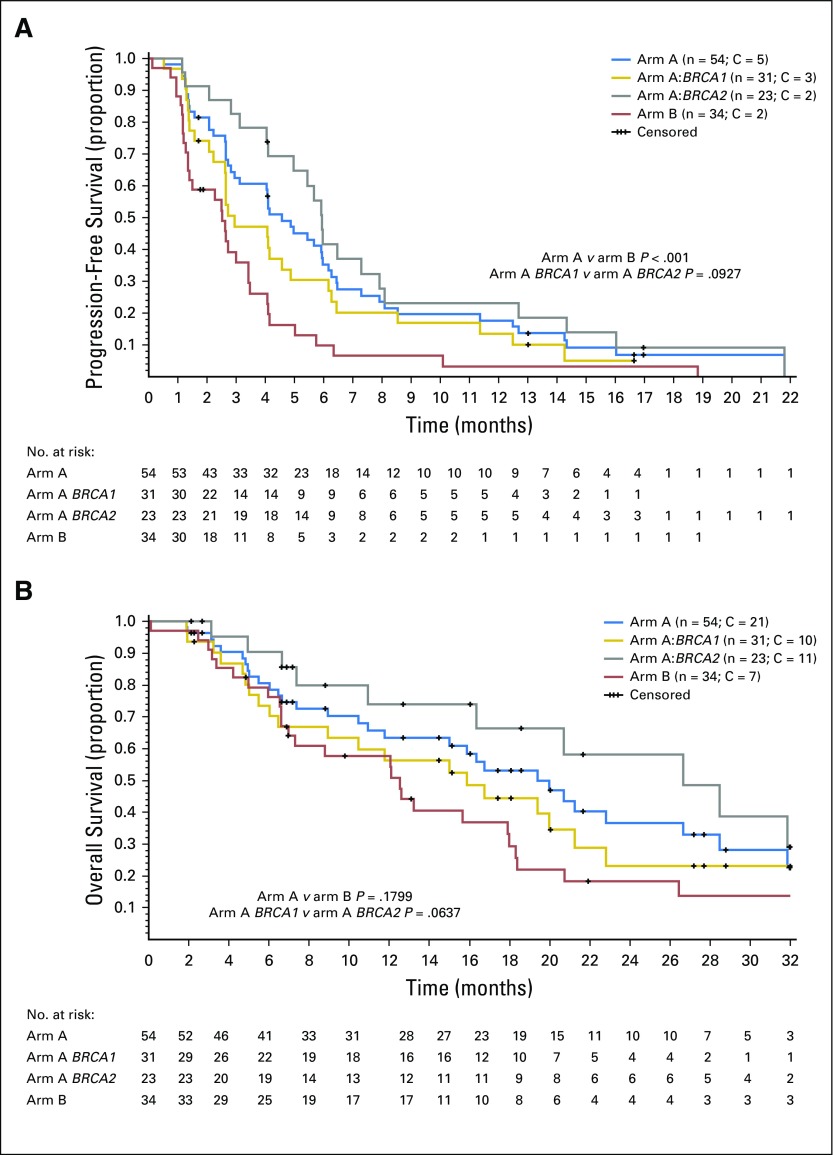
Kaplan-Meier curves for (A) progression-free survival and (B) overall survival. Included are curves for subgroups according to *BRCA* status in arm A (*BRCA1/2* mutated). C, censored.

Median PFS was 4.6 months (95% CI, 3.0 to 6.0 months [*BRCA2*, 5.9 months; *BRCA1*, 3.0 months]; Fig 3A). Median OS was 20.0 months (95% CI, 11.8 to 26.6 months [*BRCA2*, 26.6 months; *BRCA1*, 15.9 months]; Fig 3B).

### Efficacy in Arm B (*BRCA1/2* Wild-Type or Unknown Status)

Futility analysis conducted on the first 30 evaluable patients showed two objective responses (below the minimum of three responses required for recruitment extension), and this cohort was closed. The ORR in 34 total evaluable patients was 9% (95% CI, 2% to 24%), including three PRs (Table 2; Appendix Fig A1). Median duration of response was 5 months (95% CI, 2 to 18 months). Disease control rate and clinical benefit rate were 59% and 26%, respectively. Germline *BRCA1/2* status of the three patients with PRs was wild type. No other candidate genes for sensitivity were found in the pharmacogenomic substudy of tumor samples (Appendix Table A2, online only). Median PFS was 2.5 months (95% CI, 1.3 to 3.4 months; Fig 3A), and median OS was 12.5 months (95% CI, 6.6 to 17.9 months; Fig 3B).

### Safety

All 89 treated patients were evaluable for safety (Table 3). Severe AEs and laboratory abnormalities occurred at lower incidences after dose adjustment according to BSA in arm A. Grade 4 hematologic abnormalities (mainly neutropenia and thrombocytopenia) and febrile neutropenia (from 29% to 5% of patients) were notably reduced. The most common nonhematologic AEs were nausea and fatigue (74%).

**Table 3.**
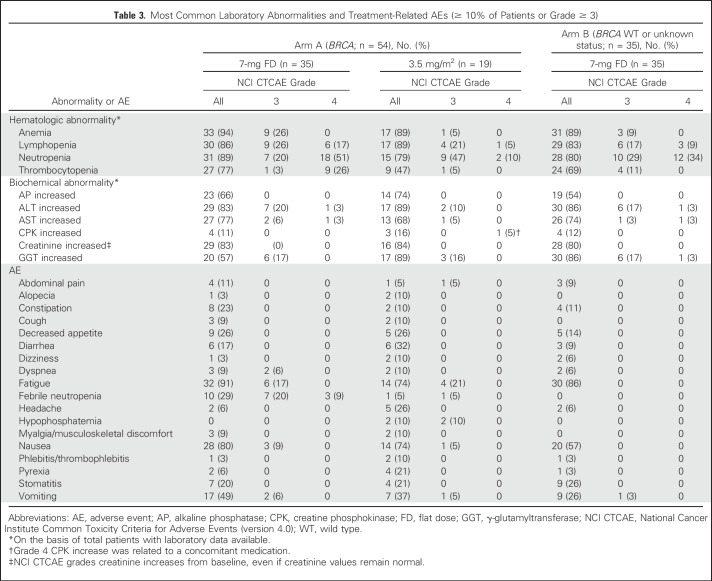
Most Common Laboratory Abnormalities and Treatment-Related AEs (≥ 10% of Patients or Grade ≥ 3)

Treatment-related discontinuations occurred in three patients treated before the amendment that adjusted dose to BSA (3.5 mg/m^2^): one in arm A (because of grade 3 dyspnea with multiple pleural metastases at baseline, talc pleurodesis, and pericardial window) and two in arm B (because of grade 3 pneumonitis and grade 3 failure to thrive/vomiting). Furthermore, one patient in arm B who was treated with the 7-mg FD died during cycle 1 as a result of septic shock with unknown relationship, which was concomitant with treatment-related grade 4 ALT/AST increase (extensive miliary liver metastases and grade 2 ALT/AST increase were present at baseline; Fig 1).

### Exome Sequencing Analysis

**Fig 4.**
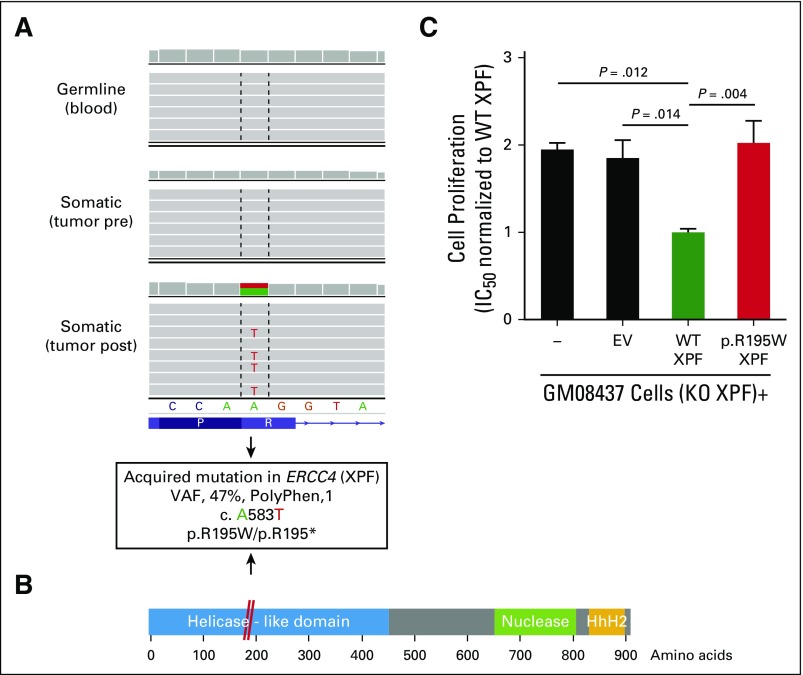
Genomic and functional validation of an acquired mutation in *ERCC4* (XPF) as a resistance mechanism to lurbinectedin. (A) Integrative genomics viewer plot shows mutation in *ERCC4* identified by exome sequencing in patient 2. (B) Representation of XPF domains that shows the location of the p.R195W mutation (red). (C) Functional validation of the p.R195W mutation in XPF-deficient (XPF knockout [KO]) GM08437 cells complemented with empty vector (EV; control), wild-type (WT), or p.R195W XPF; the IC_50_ values of lurbinectedin normalized to the levels of XPF WT are shown. Data are mean ± SEM of at least three independent experiments with three biologic replicates per group. HhH2, two consecutive helix-hairpin-helix motifs; PolyPhen, Polymorphism Phenotyping; post, postlurbinectedin; pre, prelurbinectedin; VAF, variant allele frequency.

**Fig A3.**
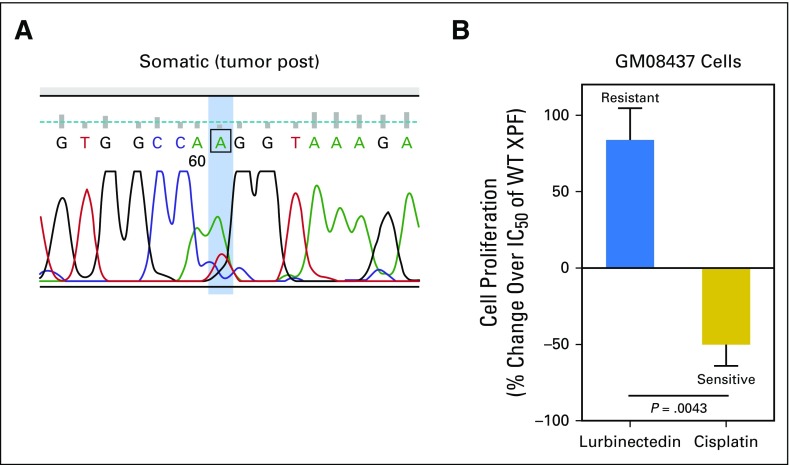
Mutation in *ERCC4* in patient 2 from arm A and functionality of XPF. (A) Sanger sequencing confirmation of the somatic *ERCC4* mutation (c.A583T, XPF p.R195W) acquired in the postlurbinectedin sample (post). (B) Differential effect of XPF deficiency on the activity of lurbinectedin and cisplatin in cell proliferation (IC_50_). Experiment performed with XPF-deficient GM08437 cells compared with wild-type (WT) XPF-complemented cells. XPF-deficient cells are resistant to lurbinectedin but sensitive to cisplatin.

A parallel unicenter translational research study included whole-exome sequencing on five paired pre- and postlurbinectedin fresh biopsy samples that met sufficient cellularity (n = 10; Appendix Table A3, online only). No secondary *BRCA1/2* mutations were identified at disease progression (PD). Because prior in vitro data reported transcription-coupled NER gene mutations as a mechanism of resistance to trabectedin,^4^ this analysis was focused on these and other DNA repair genes. An acquired mutation in the NER gene *ERCC4* (c.A583T; variant allele frequency [VAF], 47%) appeared in the postlurbinectedin sample from long-responder patient 2 (PFS, 14.3 months; Figs 4A and 4B; Appendix Fig A3A, online only). *ERCC4* encodes the nuclease XPF involved in DNA damage accumulation by lurbinectedin.^5^ The c.A583T change affects a splicing donor site, which could generate a premature stop codon and protein truncation (XPF p.R195*; Fig 4B). Alternatively, the c.A583T variant could encode a missense amino acid substitution (XPF p.R195W), the functional effect of which was evaluated in vitro. Complementation of XPF-deficient human fibroblasts (GM08437) with wild-type XPF sensitized these cells to lurbinectedin, whereas complementation with XPF p.R195W behaved as the empty vector (Fig 4C). Similarly, two somatic mutations in NER genes were identified in another patient at PD (patient 4; PFS, 5.9 months): *XPA* (p.Q216E; VAF, 38%) and *GTF2H5* (p.C12R; VAF, 29%; Appendix Table A3). Copy number variation analysis of PDX252 derived from patient 6 at lurbinectedin progression identified a complete loss of NER gene *ERCC8/CSA*, a previously reported mechanism of trabectedin resistance.^4^ In the remaining two patients with paired biopsy samples, exome sequencing did not reveal NER mutations at resistance.

**Fig A4.**
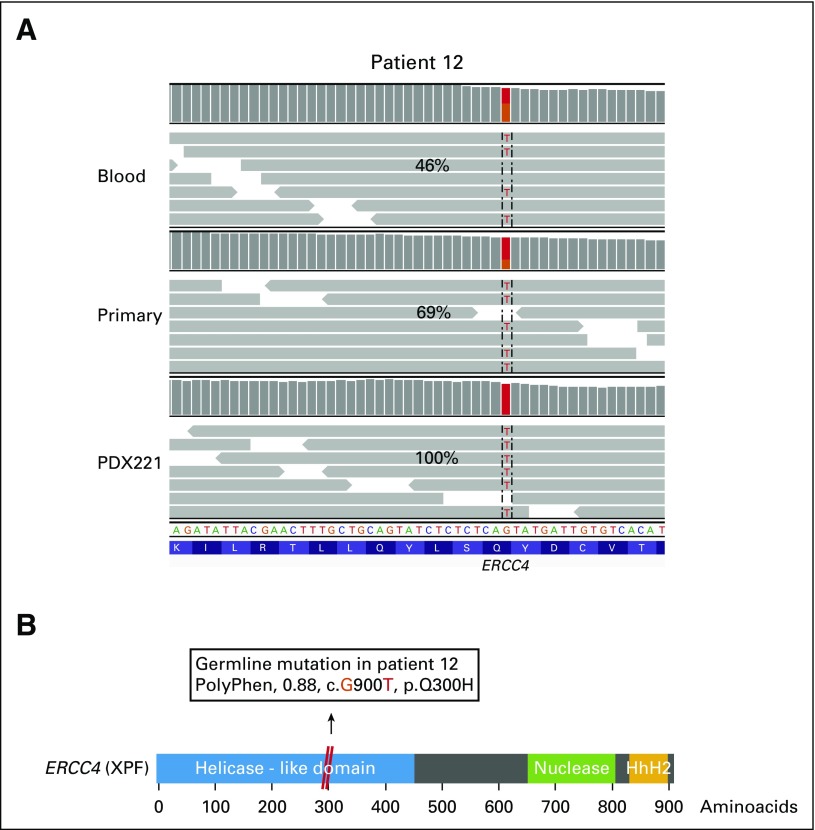
*ERCC4* mutation in patient 12 from arm A as a mechanism of primary resistance to lurbinectedin. (A) Plots of the Integrative Genomics Viewer that depict the presence (variant allele frequency [VAF]) of the missense mutation c.G900T (XPF p.Q300H; Polymorphism Phenotyping [PolyPhen] score, 0.884; possibly damaging) in patient 12 and patient-derived xenograft (PDX) model from the patient’s primary tumor (PDX221). The *ERCC4* mutation is heterozygous in blood (germline) and becomes homozygous in the primary tumor. (B) Representation of XPF domains that shows the location of p.Q300H mutation (red) in the helicase-like domain of XPF. This domain is critical for its function. HhH2, two consecutive helix-hairpin-helix motifs.

Secondary *BRCA1/2* mutations and NER-related alterations also were searched in nonpaired fresh tumor samples and in all archival tumor samples available (n = 7 primary; n = 5 metastatic). No secondary *BRCA1/2* mutations were found. A heterozygous germline mutation in *ERCC4* (XPF p.Q300H; VAF, 46%) was identified in patient 12, who had primary lurbinectedin resistance. Of note, this mutation was enriched to homozygosity in the primary tumor (VAF, 69%) and in the metastatic relapse (VAF, 60%; Appendix Figs A4A and A4B, online only). None of the long-responder patients carried an *ERCC4* mutation. A mutation in the XPF scaffold protein SLX4/FANCP was found in patient 10, who showed stable disease (PFS, 2.7 months; p.A952M; VAF, 91%). Thus, these results suggest that NER-related alterations may arise as a mechanism of resistance to lurbinectedin.

### In Vivo Studies With Lurbinectedin and Cisplatin

**Fig A5.**
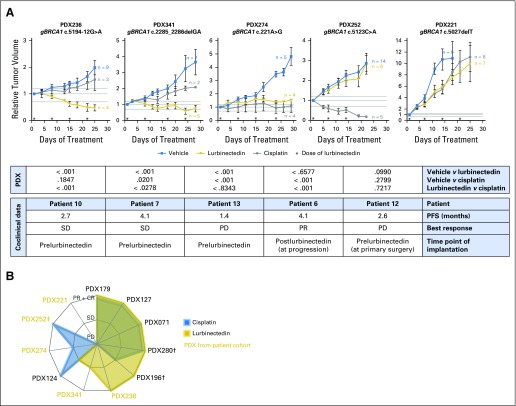
Antitumor activity of lurbinectedin and cisplatin in vivo. (A) Plots depict the change in tumor volume upon treatment with vehicle, lurbinectedin, or cisplatin in five patient-derived xenograft (PDX) models from patients in arm A. Dotted lines represent the boundaries of disease progression (PD; > 20%) and partial response (PR; < 30%), being stable disease (SD) in between. The number of tumors per group also are shown. Error bars are SEM. The table lists statistical comparisons among treated PDX groups (*P* value is shown), and coclinical information of the patient of each PDX. (B) Radar plot of the response to lurbinectedin and cisplatin in 11 PDX models derived from patients with germline *BRCA1/2* mutations and either breast or ovarian cancer, including the five PDXs shown in (A). Filled area represents sensitivity to the drugs (ie, SD, PR, CR, PD). PDX from patients in arm A are shown in gold. PDX252 was derived from patient 6 at progression to lurbinectedin (note that the resulting PDX recapitulated resistance to lurbinectedin). (†) PDX from BRCA1-mutated ovarian cancer. (‡) PDX obtained at lurbinectedin progression.

Prior data suggested that NER alterations could induce resistance to lurbinectedin but increase sensitivity to cisplatin,^4,5^ and we confirmed this in vitro (Appendix Fig A3B, online only). Antitumor activity of cisplatin and lurbinectedin was investigated in five coclinical PDX models derived from patients in arm A (Appendix Table A3; Appendix Fig A5A, online only). All PDX models recapitulated the clinical response to lurbinectedin. Three of five models showed opposite responses for lurbinectedin and cisplatin. Of note, one PDX (PDX252) generated at progression to lurbinectedin harbored an *ERCC8/CSA* loss and responded to cisplatin. Six additional PDX models from a collection of *BRCA1/2*-mutated PDXs^12^ were tested, and the spectrum of activity of lurbinectedin and cisplatin only partially overlapped (Appendix Fig A5B,online only).

**Fig A6.**
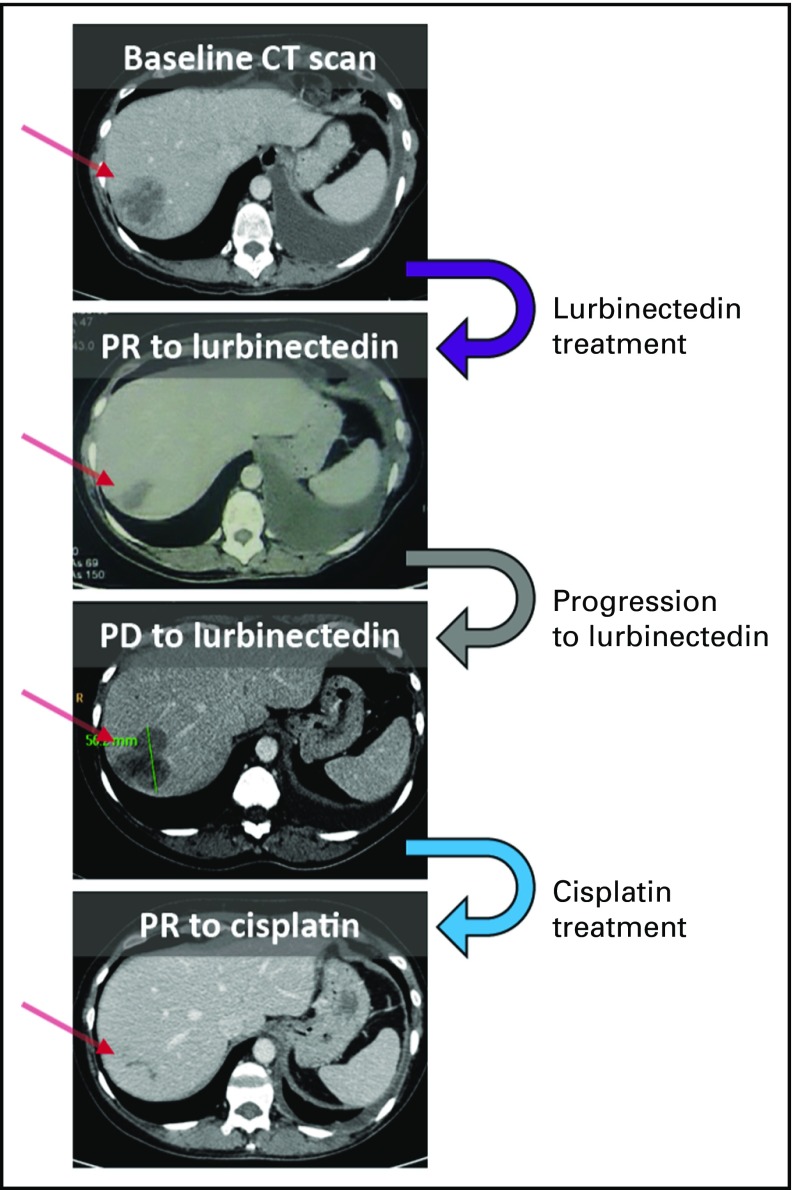
Sequential radiologic responses to lurbinectedin and cisplatin in patient 4. Mutations in the nucleotide excision repair (NER) genes *XPA* and *GTF2H5* were acquired at progression to lurbinectedin. A confirmed partial response (PR) to cisplatin was observed after lurbinectedin progression. Computed tomography (CT) scans are shown. The red arrow points to the liver metastasis. PD, disease progression.

Supporting evidence for clinical cisplatin sensitivity after progression to lurbinectedin is exemplified in Appendix Fig A6 (online only). The patient who harbored mutations in *XPA* and *GTF2H5* in the liver metastasis at lurbinectedin progression was subsequently treated with platinum-based chemotherapy and achieved a sustained PR.

## DISCUSSION

This phase II trial met its primary end point and showed lurbinectedin to be active in patients with *BRCA1/2*-mutated MBC. Overall, 22 of 54 patients with *BRCA1/2* mutations achieved a confirmed response (ORR, 41%), including two CRs, with a median duration of response of 6.1 months (range, 3 to 11 months). Median PFS and OS were 4.6 and 20.0 months, respectively.

The most common nonhematologic (nausea and fatigue) and hematologic (neutropenia) AEs observed in patients with MBC were already reported in a phase I study conducted at 7 mg FD^11^ and in a phase II study in patients with platinum-resistant/refractory advanced ovarian cancer.^13^ Nonetheless, the lurbinectedin safety profile was improved after implementation of BSA-based dosing. Severe events occurred at lower incidences, with a notable reduction in the rate of grade 4 hematologic abnormalities and febrile neutropenia. Cumulative toxicity was not observed, and most patients achieved long-time exposure. Therefore, lurbinectedin 3.5 mg/m^2^ seems to be a safe and active dose for future trials.

The most remarkable antitumor activity was found in patients with *BRCA2* mutation (ORR, 61%; PFS, 5.9 months; OS, 26.6 months) in whom ORR increased to 72% and 71% in those without prior PARPi and platinum therapy, respectively. Trabectedin also had shown higher efficacy in patients with *BRCA2* MBC versus *BRCA1* (ORR, 33% *v* 9%; median, PFS 4.7 *v* 2.5 months).^3^ ORR for PARPi therapy in patients with *BRCA*2 MBC in phase I/II trials was 22% with olaparib,^14^ 36% with veliparib,^15^ and 34% with talazoparib.^16^ The reasons for the different efficacy of lurbinectedin in *BRCA2* versus *BRCA1* disease are under investigation. In addition to its well-known role in HRR, *BRCA2* prevents the formation of RNA-DNA hybrids (R-loops) that occurs during the elongation step of transcription by RNA polymerase II.^6,7^ One hypothesis to explain the differential activity of trabectedin and lurbinectedin observed in *BRCA2*- compared with *BRCA1-*mutated MBC is the concurrence of deficient DNA repair and the formation of R-loops. Recognition of HRR deficiency as a biomarker of sensitivity to platinum agents has led to their reconsideration for the treatment of *BRCA1/2* mutation–associated tumors. Indeed, the Triple-Negative Breast Cancer Trial showed germline *BRCA1/2* tumors to be more sensitive to carboplatin than docetaxel and provided clinical evidence to treat these patients with platinum in the metastatic setting.^17^ Nevertheless, previous platinum exposure decreases the benefit to other *BRCA1/2*-directed therapies, such as PARPi.^16,18^ In this regard, in a phase II study with talazoparib, patients who responded to platinum and did not progress until at least 8 weeks after treatment had an ORR of 21% versus 37% among those who were platinum naïve in the metastatic setting.^16^ In the phase III OlympiAD^18^ trial with olaparib, the ORR (unconfirmed response) decreased from 66% in platinum-naïve patients to 46% in patients with prior platinum therapy and no progression during treatment. Responses to lurbinectedin also decreased in patients previously exposed to platinum, although comparisons with PARPi trials^14,16^ cannot be made because of different inclusion criteria in the platinum-free interval. Although clinical data show decreased sensitivity to lurbinectedin in platinum-pretreated patients, preclinical data in PDXs suggest a partial overlap of efficacy and resistance mechanisms. Additional research is needed to delineate the most appropriate therapeutic sequence to minimize cross-resistances.

Secondary mutations in *BRCA1* and *BRCA2* that re-establish the reading frame may restore HRR proficiency and render cancer cells resistant to agents that target DNA damage.^19^ In this study, exome sequencing showed no secondary *BRCA1/2* mutations in either primary or acquired resistance. In contrast, alterations in NER-related genes were found. Somatic mutations in *ERCC4* could explain primary resistance to lurbinectedin in a nonresponder patient and acquired resistance in another long-responder patient. VAF in two mutated genes involved in active transcription and NER *(XPA* and *GTF2H5*) increased at lurbinectedin progression in another patient. Of note, the acquisition of resistance to lurbinectedin did not preclude a subsequent response to platinum-based chemotherapy in this patient, as was also observed in a coclinical acquired-resistance model (PDX252). Limitations of this study were the small size of some of the subsets evaluated and the optional participation for the translational unicentric substudy, which resulted in a limited sampling. Additional studies will have to focus on lurbinectedin activity as a transcription inhibitor in HRR-deficient/NER-active tumors.

In conclusion, lurbinectedin has a unique mechanism of action, with promising efficacy observed in *BRCA1/2-*mutated MBC. The noteworthy specific activity of lurbinectedin in patients with *BRCA2* mutation warrants additional clinical development.

## Figures and Tables

**Table A1.**
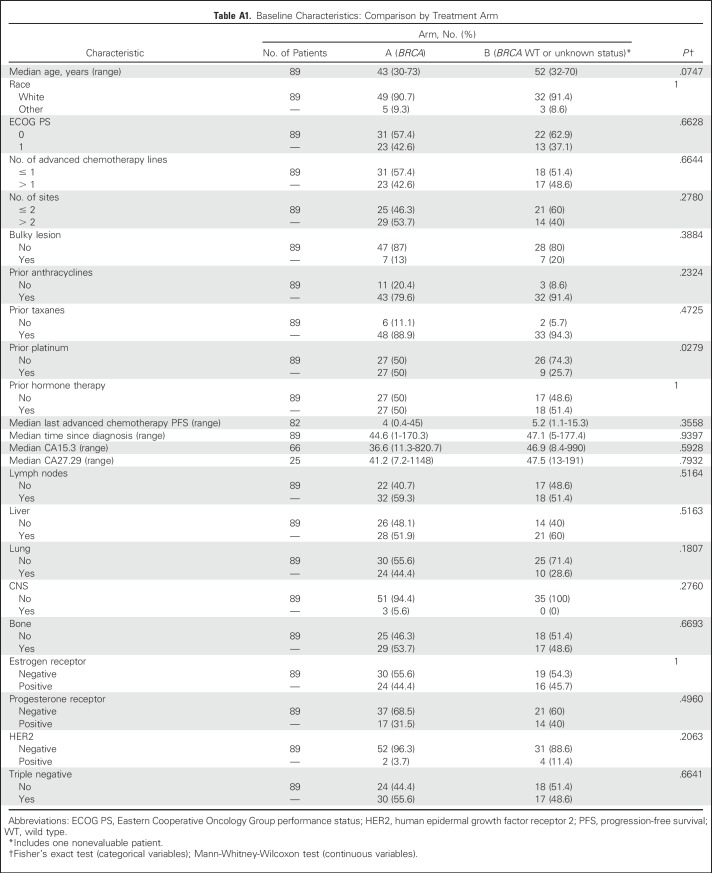
Baseline Characteristics: Comparison by Treatment Arm

**Table A2.**
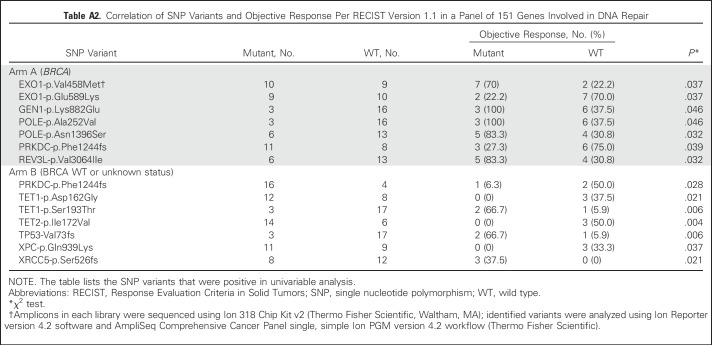
Correlation of SNP Variants and Objective Response Per RECIST Version 1.1 in a Panel of 151 Genes Involved in DNA Repair

**Table A3.**
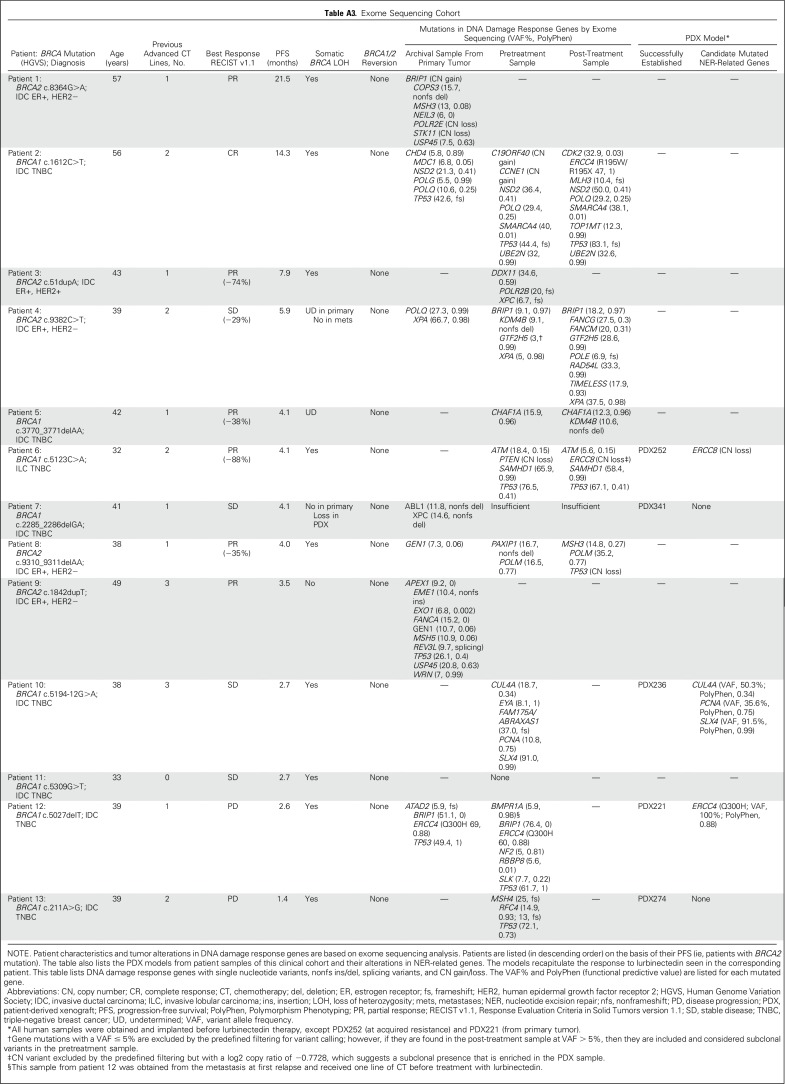
Exome Sequencing Cohort

## Appendix

### Methods

#### Pharmacogenomic substudy.

Patients optionally participated in this substudy (52 patients; total of 39 samples). A customized cancer panel was used to obtain the exon sequence of genes involved in DNA repair and cancer biology. The panel comprised four primer pools that totaled 1,774 primer pairs (target size, approximately 2.5 Mb). Genomic DNA was extracted from macrodissected formalin-fixed paraffin-embedded (FFPE) tumor tissue and quantified by Qubit (Thermo Fisher Scientific, Waltham, MA); its quality was determined by ladder polymerase chain reaction (PCR) using 2 ng of genomic DNA (gDNA). A library was obtained with each of the four primer pools using 10 ng of gDNA, and the amplicons in each library were sequenced using Ion 318 Chip Kit v2 (Thermo Fisher Scientific). Identified variants (both in exonic regions and in noncoding regions) were analyzed using Ion Reporter version 4.2 software and AmpliSeq Comprehensive Cancer Panel using a single, simple Ion PGM version 4.2 workflow (Thermo Fisher Scientific). Potentially deleterious variants were predicted using the ANNOVAR software tool (Wang et al: Nucleic Acids Res 38:e164, 2010), and single nucleotide polymorphisms were identified using Ion Reporter version 4.2 software.

#### Fresh biopsy sample cohort.

All participants from one of the investigational sites (Vall d’Hebron University Hospital, Barcelona, Spain) were asked to undergo voluntarily a fresh core biopsy. The sample was taken from a metastatic lesion before starting treatment with lurbinectedin. Participants signed local institutional review board–approved consent forms, unless a representative metastatic sample was already available. A paired biopsy sample of the same tumor lesion from patients who achieved a Response Evaluation Criteria in Solid Tumors (RECIST) version 1.1 response was obtained at the time of progression to lurbinectedin, if amenable. Exome sequencing analysis and xenograft implantation was done from these biopsy samples.

#### Exome sequencing.

Patients provided written informed consent for somatic and germline DNA analysis. Fresh frozen tumor samples, along with a blood sample, were subjected to whole-exome sequencing. Additional archival samples from the same patient cohort were sequenced when available. Samples initially were assessed for tumor content on the basis of a hematoxylin-eosin stain. gDNA was extracted from frozen samples with the QIAamp DNA Mini Kit (QIAGEN, Hilden, Germany), from archival FFPE samples with the Maxwell 16 FFPE Plus LEV DNA Purification Kit (Promega, Madison, WI), and from whole blood with the QIAamp DNA blood Mini Kit (QIAGEN) according to the manufacturers’ instructions. Library preparation was performed while following the standard Illumina protocol (Genomic DNA Sample Prep Kit; Illumina, San Diego, CA). One microgram of DNA was fragmented; ends repaired; and an adenine ligated to each of the 3′ ends, where sample-specific adaptors were linked. Libraries were amplified using eight cycles of PCR, and exome enrichment was performed using specific biotinylated probes (SureSelect XT Human All Exon 50 Mb; Agilent Technologies, Santa Clara, CA). After enrichment, the exome libraries were PCR amplified (eight cycles), quantified, and loaded in a HiSeq 2000 sequencing system (Illumina).

A quality check of the raw data were performed by the FastQC tool (www.bioinformatics.babraham.ac.uk/projects/fastqc). Paired-end 100-base sequences were aligned to the Sanger human reference genome (hg19) using the Burrows-Wheeler Alignment tool version 7.12 (Li et al: Bioinformatics 26:589-595, 2010). This yielded a depth of coverage in targeted regions of approximately 70%, with approximately 70% of the exome having at least 10 reads. The resulting binary alignment map files were processed using SAMtools version 1.19 (Li et: Bioinformatics 25:2078-2079, 2009; Li: Bioinformatics 27:2987-2993, 2011) and the Genome Analysis ToolKit version 3.2.0 (McKenna et al: Genome Res 20:1297-1303, 2010). Mutations were called with VarScan2 version 2.3.7 software (Koboldt et al: Genome Res 22:568-576, 2012) using the following parameters: somatic, minimum 8× coverage, minimum five reads supporting the variant allele, minimum variant allele frequency (VAF) of 5%, strand-bias filtering, and *P* < .05. Annotation of the variant call format files was performed with ANNOVAR software (Wang et al: Nucleic Acids Res 38:e164, 2010). Except for germline *BRCA1* and *BRCA2*, variants were filtered on the basis of being annotated as exonic by the RefSeq release 45 database (National Center for Biotechnology Information, Bethesda, MD) and nonsynonymous. Final manual review was done to check for local indel misalignments and homopolymer false-positive calls. Copy number variations (CNVs) were identified using the tool SeqGene (Deng: BMC Bioinformatics 12:267m 2011) and the R Bioconductor DNAcopy package (Venkatraman et al: Bioinformatics 23:657-663, 2007). We focused on mutations and CNVs of genes involved in DNA damage repair on the basis of an in-home–made list of 485 genes.

#### Implantation of patient-derived xenograft models and in vivo experiments.

Fresh tumor samples from patients were prospectively collected and immediately implanted into the lower flank of nude mice under an institutional review board–approved protocol and the associated informed consent (Bruna et al: Cell 167:260-274, 2016). Upon growth of the engrafted tumors, the model was perpetuated by serial transplantation onto the lower flank of Naval Medical Research Institute nude mice. To evaluate the sensitivity to the drugs, tumor-bearing mice were equally distributed into treatment groups, with tumors ranging in size from 100 to 300 mm^3^. Lurbinectedin (0.18 mg/kg) and cisplatin (6 mg/kg) were administered intravenously weekly unless relative tumor volume < 0.5 or weight loss > 20%. Tumor growth was measured with caliper biweekly since the first day of treatment, and tumor volume was calculated as V = 4π / 3 / L × l^2^, where L is the largest diameter and l is the smallest. The antitumor activity was determined by comparing tumor volume after three to four cycles or at best response (in sensitive patient-derived xenograft [PDX], minimum value of percent tumor volume change sustained for at least 10 days) to its baseline: percent tumor volume change = (V – V_initial_) / V_initial_ × 100. To classify the response of the subcutaneous implants, we modified the RECIST to be based on the percent tumor volume change: complete response, best response < −95%; partial response, best response < −30%; stable disease −30% < best response < 20; and disease progression, best response > 20%. Experiments were conducted using the European Union animal care directive (2010/63/EU) and were approved by the Ethical Committee of Animal Experimentation of the Vall d’Hebron Research Institute.

### Statistical Methods: Information From the Phase II Trial

Sample size was calculated using East version 5.4 software (Cytel, Cambridge, MA) for each arm as a single proportion test, including a futility analysis in each one. The next hypotheses were selected as follows:

- Arm A (*BRCA*): At least 53 evaluable patients had to be recruited to test the null hypothesis that objective response rate (ORR) was ≤ 20% (*P* ≤ .20) versus the alternative hypothesis that ≥ 40% patients have an objective response (*P* ≥ .4). With these assumptions, if the number of evaluable patients with an objective response is ≥ 17, then this would allow the rejection of the null hypothesis.
- Arm B (*BRCA* wild-type or unknown status): At least 64 evaluable patients had to be recruited to test the null hypothesis that ORR is ≤ 10% (*P* ≤ .10) versus the alternative hypothesis that ≥ 25% patients have an objective response (*P* ≥ .25). With these assumptions, if the number of evaluable patients with an objective response is ≥ 12, then this would allow the rejection of the null hypothesis.

The variance of the standardized tests was based on the null hypothesis. The type I error (α) associated with each one-sided test was 0.025, and the type II error (β) was < 0.1; hence, statistical power was > 90%.

Futility analysis controlled by the gamma family boundary was performed when 20 and 30 patients had been evaluated in arms A [gamma(−2)] and B [gamma(−1.5)], respectively. If fewer than four of 20 patients in arm A or fewer than three of 30 patients in arm B achieved an objective response, recruitment in that arm had to be stopped.
